# Telomere-to-telomere assembled and centromere annotated genomes of the two main subspecies of the button mushroom *Agaricus bisporus* reveal especially polymorphic chromosome ends

**DOI:** 10.1038/s41598-020-71043-5

**Published:** 2020-09-04

**Authors:** Anton S. M. Sonnenberg, Narges Sedaghat-Telgerd, Brian Lavrijssen, Robin A. Ohm, Patrick M. Hendrickx, Karin Scholtmeijer, Johan J. P. Baars, A. van Peer

**Affiliations:** 1 Plant Breeding, Wageningen University and Research, Droevendaalsesteeg 1, 6708 PB Wageningen, The Netherlands; 2 Ceradis B.V., Agro Business Park 10, 6708 PW Wageningen, The Netherlands; 3 Department of Microbiology, University of Utrecht, Padualaan 8, 3584 CH Utrecht, The Netherlands

**Keywords:** Computational biology and bioinformatics, Genetics

## Abstract

*Agaricus bisporus*, the most cultivated edible mushroom worldwide, is represented mainly by the subspecies var. *bisporus* and var. *burnettii*. var. *bisporus* has a secondarily homothallic life cycle with recombination restricted to chromosome ends, while var. *burnettii* is heterothallic with recombination seemingly equally distributed over the chromosomes. To better understand the relationship between genomic make-up and different lifestyles, we have de novo sequenced a *burnettii* homokaryon and synchronised gene annotations with updated versions of the published genomes of var. *bisporus*. The genomes were assembled into telomere-to-telomere chromosomes and a consistent set of gene predictions was generated. The genomes of both subspecies were largely co-linear, and especially the chromosome ends differed in gene model content between the two subspecies. A single large cluster of repeats was found on each chromosome at the same respective position in all strains, harbouring nearly 50% of all repeats and likely representing centromeres. Repeats were all heavily methylated. Finally, a mapping population of var. *burnettii* confirmed an even distribution of crossovers in meiosis, contrasting the recombination landscape of var. *bisporus*. The new findings using the exceptionally complete and well annotated genomes of this basidiomycete demonstrate the importance for unravelling genetic components underlying the different life cycles.

## Introduction

The last decade has shown a sharp increase in the number of sequenced fungal genomes. Recently, the genome sequence of 90 mushrooms has been published^1^, but especially the ongoing 1,000 Fungal Genomes Project (https://1000.fungalgenomes.org/home/) will generate an extensive insight into the genomes of a wide range of fungal families and species. However, most genome sequences typify only one strain of a species, do not represent telomere-to-telomere chromosomes, do not have an accurate annotation of transposable elements, and centromeres remain unidentified. Especially complete and well annotated genomes of subspecies, with different life cycles, are a prerequisite to study the relation between genomic content and effects on different life cycles. A good example of a species with different life cycles is the button mushroom *Agaricus bisporus*, one of the most cultivated edible mushrooms in the world. It is an amphithallic basidiomycete represented mainly by two subspecies, i.e., *Agaricus bisporus* var. *bisporus* and *A. bisporus* var. *burnettii*. All commercial varieties and most wild isolates are represented by the subspecies var. *bisporus*. Despite a few beneficial traits such as resistance to some diseases^2,3^, the average quality of var. *burnettii* strains is poor^4^. Especially the production of abundant numbers of small mushrooms that show a quick maturation (cap opening) and asymmetric caps are considered as poor quality. The var. *bisporus* will therefore remain the dominant commercial variety, and thus the platform for breeding for the time being. While the former subspecies has a mainly secondary homothallic life cycle where basidia produce two spores, var. *burnettii* is mainly heterothallic and has four-spored basidia^5–7^. Previous research has also shown that the two subspecies differ in distribution of meiotic recombination over the chromosomes. In var. *bisporus*, crossovers are mainly restricted to chromosome ends^8^. In contrast, linkage maps generated with offspring of var. *bisporus* × var. *burnettii* indicated that *burnettii* shows a more even distribution of crossovers over the chromosomes^9^. The cumbersome isolation of homokaryotic offspring (only few % of spores are haploid) and especially the restriction of recombination to chromosome ends hampers an efficient breeding of var. *bisporus*. The availability of complete and well annotated genomes of both subspecies can help to unravel the genetic components underlying the differences in basidial spore number and recombination landscape. The trait basidial spore number (BSN) has been mapped previously on chromosome 1^9^. The genetic base for both phenotypes, however, are unknown. High quality, well annotated, telomere-to-telomere genomes sequences, are a prerequisite to unravel the genes involved in these traits, especially for the recombination landscape since one is studying the effect of crossovers on the changes in the genome during meiosis.

Previously, high quality genome sequences have been published for two homokaryons of var. *bisporus* and a var. *burnettii* homokaryon^8,10^. The latter has, however, a low quality with 2016 scaffolds representing 13 chromosomes. In this study, we generated also a high quality whole genome assembly of the var. *burnettii*. We recovered both constituent homokaryons of a *burnettii* heterokaryon, de novo sequenced one homokaryon and resequenced both. Previously, an impression of the recombination landscape of var. *burnettii* was obtained indirectly using offspring of a cross between a *bisporus* and a *burnettii* homokaryon^9^. So far, no recombination was examined in offspring of a *burnettii* variety. We generated here a linkage map from an offspring of this *burnettii* heterokaryon giving for the first time a direct impression of the recombination landscape of this subspecies. The linkage map was also used to assemble the de novo genome of the *burnettii* homokaryon into 16 scaffolds. Ten chromosomes are single scaffolds whereas the other six scaffolds could be assigned to the three remaining chromosomes, and all except three telomeres were recovered. The gene predictions of the previously published whole genomes of the var. *bisporus* homokaryons and the *burnettii* homokaryon were updated, generating a consistent set of gene predictions across these homokaryons. Special attention was given to the annotation of transposable elements and other repeats. Each chromosome of all three homokaryons has a large heavily methylated repeat cluster likely representing a centromere. Approximately half of the sequences of the putative centromeres consist of repeats specific to these putative centromeres and are not found elsewhere in the genome. To our knowledge these are the first complete annotated centromeres of basidiomycetes. The availability of three high quality telomere-to-telomere genomes representing two subspecies is unique and revealed especially polymorphism in the chromosome ends between the two subspecies.

## Results

### Genome sequences

**Figure 1:**
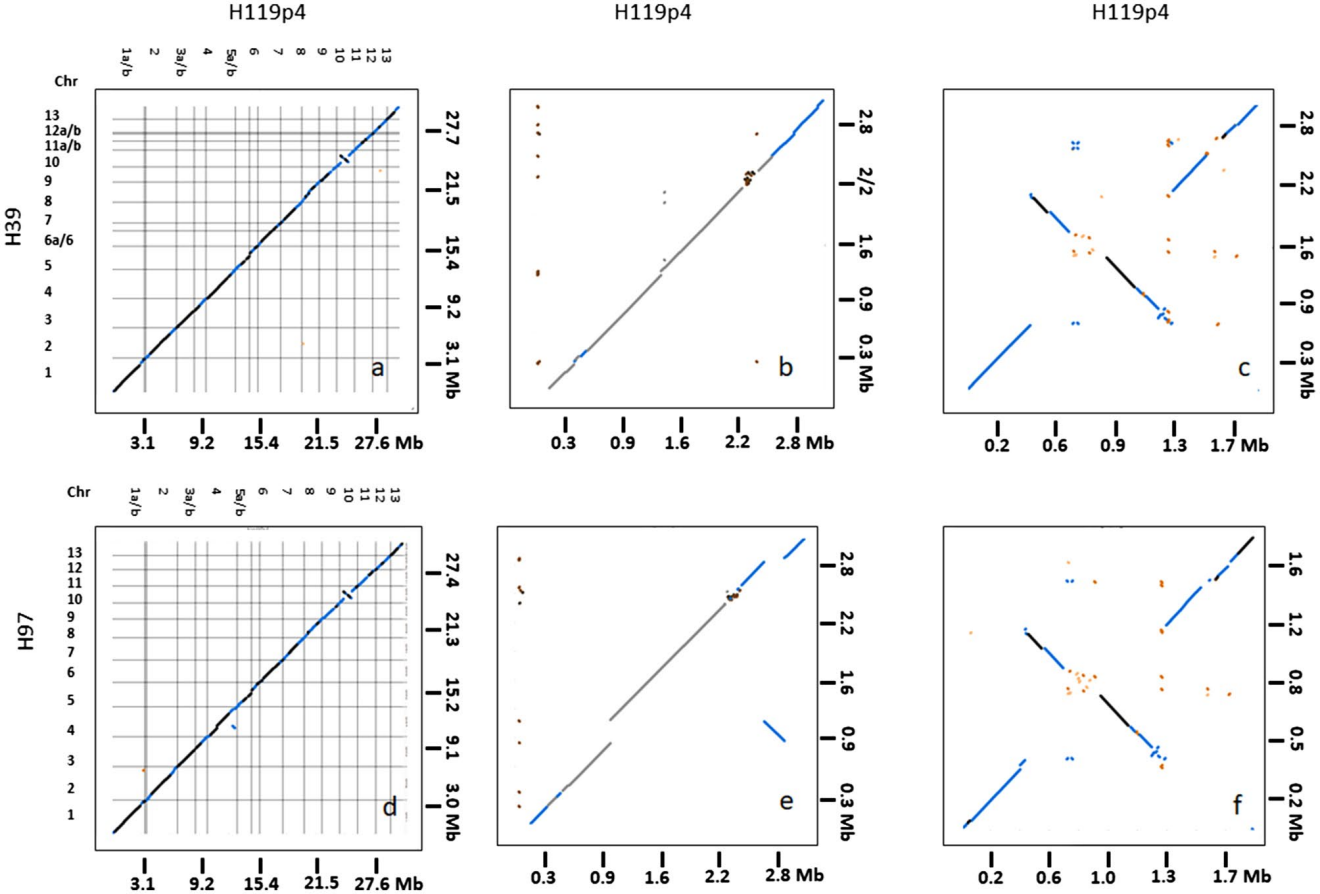
Dot-plot comparison between the genomes of the var. *bisporus* homokaryons H39 and H97 against var. *burnettii* H119p4. (**A**,**D**) Mummer 3.23 (nucmer) plots of whole genomes (13 chromosomes) of H39 (A) and H97 (**D**) against homokaryon H119p4. (**B**,**E**) plots of chromosome 4 of H39 (**B**) and H97 (**E**) against H119p4; an inversion (blue) is seen in H97 compared to H119p4. (**C**,**F**) plots of chromosome 10 of H39 (**C**) and H97 (**F**) against H119p4; An inversion is seen in H39 and H97 compared to H119p4. The figure was drawn using D-Gernies (https://dgenies.toulouse.inra.fr/).

To generate a high quality WGS of a *burnettii* variety, heterokaryon 119/9 (JB-13), originating from the *Agaricus* Recovery Program, was used^11^. Both constituent homokaryons were recovered by protoplasting and designated as H119p1 and H119p4. H119p4 was de novo sequenced with PacBio RSII and both H119p1 and H119p4 were resequenced using Illumina HiSeq. A set of 154 homokaryotic single spore isolates (SSI) of the heterokaryon 119/9 were isolated and genotyped, together with the parental homokaryons, using Sequenced Based Genotyping (SBG; see paragraph 2.3). The SBG sequencing data were used to generate a linkage map, helpful to assemble the de novo PacBio data of H119p4 into a complete genome of 16 scaffolds with genome size of 30.7 Mb. This size is very similar to those of the var. *bisporus* homokaryons H39 and H97^8^. Chromosomes 1, 3 and 5 each consisted of 2 non-overlapping scaffolds. All other chromosomes were complete or nearly complete, i.e. consisting of one scaffold. For chromosomes 3, 9, and 12 the telomere sequence on one side was missing (Tables 1, S1 for sequence statistics). Next, we performed a comparative analysis of the genome assemblies using MUMmer. All strains were compared to each other in a pairwise manner. The alignment of all three genomes revealed that they are highly collinear (Fig. 1). One large inversion of ∼ 800 Kb was found on chromosome 10 of H119p4 compared to the same chromosome of the *bisporus* varieties, indicating a structural difference between the var. *bisporus* homokaryons and the var. *burnettii* homokaryon. A smaller inversion of ∼ 250 Kb was found on chromosome 4 of H119p4 compared to the H97 genome. This inversion was not seen relative to the H39 genome and might be a real difference or due to a miss-assembly in the H97 genome. The appearance of the gaps or shifts in the Mummer plots viewed for all chromosomes coincides with clusters of repeats, as has been described previously^8^. Three complete genomes of genetically unrelated genotypes representing two subspecies are now available for *A. bisporus.*

**Table 1:** The assembled genomes of homokaryons H97, H39 and H119p4.

Chromosome	H97_3.1				H39_3.1				H119_p4v2.1			
	Size (bp)	5′ Telomere	3′ Telomere	Nr. of scaffolds	Size (bp)	5′ Telomere	3′ Telomere	Nr. of scaffolds	Size (bp)	5′ Telomere	3′ Telomere	Nr. of scaffolds
1	3,550,205	Yes	Yes	1	3,779,356	Yes	Yes	1	3,639,502	Yes	Yes	2
2	3,489,786	Yes	Yes	1	3,241,356	Yes	Yes	1	3,265,402	Yes	Yes	1
3	3,131,856	Yes	Yes	1	3,126,264	Yes	Yes	1	3,132,325	Yes	No	2
4	3,112,789	Yes	Yes	1	3,115,696	Yes	Yes	1	3,158,235	Yes	Yes	1
5	2,550,681	Yes	No	1	2,477,729	Yes	Yes	1	2,380,213	Yes	Yes	2
6	2,329,815	Yes	Yes	1	2,476,259	Yes	Yes	2	2,426,497	Yes	Yes	1
7	2,323,169	No	Yes	1	2,233,015	Yes	Yes	1	2,254,680	Yes	Yes	1
8	1,953,186	Yes	Yes	1	2,155,647	Yes	Yes	1	1,867,134	Yes	Yes	1
9	1,688,379	No	No	1	1,624,235	Yes	No	1	1,867,033	Yes	No	1
10	1,762,795	Yes	Yes	1	1,787,784	Yes	Yes	1	1,897,446	Yes	Yes	1
11	1,708,529	Yes	Yes	1	1,733,467	Yes	Yes	2	1,931,181	Yes	Yes	1
12	1,482,581	Yes	Yes	1	1,576,767	Yes	Yes	2	1,559,381	Yes	No	1
13	1,334,073	Yes	No	1	1,405,511	Yes	Yes	1	1,323,473	Yes	Yes	1
Total size	30,417,844				30,733,086				30,702,502			

For each genome the (estimated) size of the chromosomes, presence of telomeres and the number of scaffolds is indicated. Except for 3 chromosomes of H39 and for H119p4, all are represented by one scaffold that for most chromosomes stretches into the telomeres.

### Gene prediction and functional annotation

**Figure 2:**
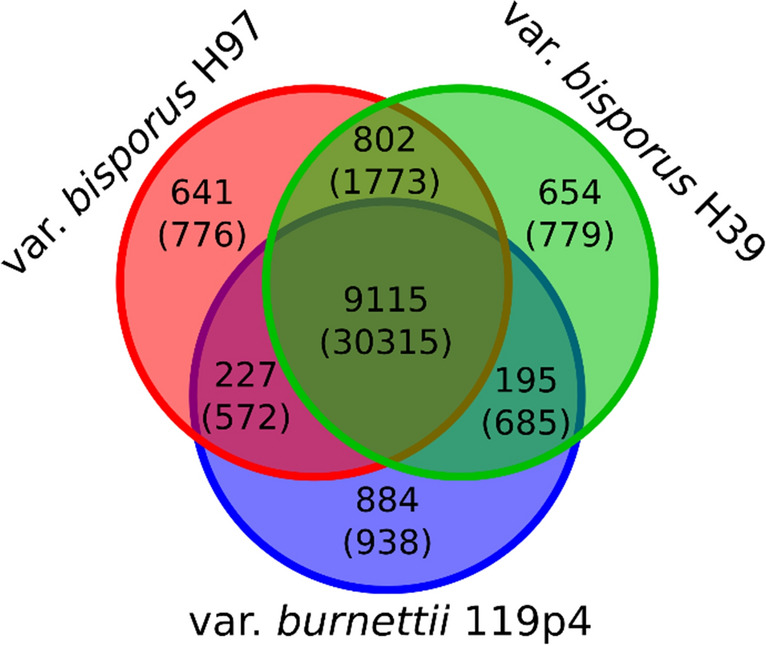
Comparative analysis of predicted proteins of three strains of *A. bisporus*. The predicted proteins were clustered into gene families using Orthofinder. The VENN diagram displays the number of gene families shared between or unique to strains. The total number of predicted proteins in these gene families are indicated between parentheses. Note that the predicted genes were not filtered for repetitive elements.

The gene prediction resulted in 11,971, 12,104 and 11,763 genes for strains H97, H39 and H119p4, respectively (Table S1). When possible, orthologs between strains were given the same gene ID. Functional annotation was performed on these genes in an effort to predict their function. For example, over 60% of the predicted genes were annotated with a PFAM domain. The quality of the set of gene predictions is demonstrated by the high values of both CEGMA and BUSCO scores (> 99%). As expected, regions encoding genes and repetitive regions are largely mutually exclusive. This is demonstrated by the low values of the Jaccard statistics for these sets: 0.11 in each of the three strains (the Jaccard statistic is a measure of overlap between two sets of genomic regions and is 0 for no overlap and 1 for complete overlap). The predicted proteins of all three strains were grouped into clusters based on homology using Orthofinder. In this context, a cluster can be interpreted as a gene family. This comparative analysis revealed that, as expected, the majority of the gene families are shared between the strains (Fig. 2). Strains H39 and H97 are more similar to each other than to H119p4, since H39 and H97 share a higher number of gene families, while strain H119p4 has the highest number of singleton families. Next, we performed a comparative analysis of the genome assemblies using MUMmer. First, all strains were compared to each other in a pairwise manner. Subsequently, genomic regions were identified that were unique to a strain, or present in two strains but absent in the third. Genes that overlapped those regions were considered unique to those combinations as well. This revealed that each strain has several genes unique compared to the other two strains. Since transposable elements represent most of the differences, all annotated repetitive elements (see paragraph 3.4) were removed from these lists. The final numbers of differences between the *burnettii* strain on the one hand and both *bisporus* strains on the other are larger than among the two *bisporus* strains, as expected (Table 2). Remarkably, 76 (one third) of the 236 unique genes in H119p4 are concentrated in subtelomeric regions, often very close to the telomeres. The only genes of the 236 unique genes with a PFAM hit are one DEAH helicase, an elongation factor of RNA pol II, and 19 gene models representing RecQ DNA helicases, the latter all in subtelomeric regions. Of these, nine represent likely full-length RecQ DNA helicases. Alignment of the 19 RecQ helicases shows that they can be separated in two groups: one group of 10 genes (three complete and seven truncated) with identical protein sequences and nine genes (seven complete, two truncated) that are similar but with more sequence variation; (Fig. S2B). We had Illumina reads available for a number of other homokaryons, two of var. *bisporus* and four of var. *burnettii*, including the other homokaryon of 119/9 (H119p1) and the JB137-S8 data from the JGI database. Screening these showed that the subtelomeric RecQ helicases are present in all five *burnettii* homokaryons and in none of the four *bisporus* homokaryons, and can thus be considered as specific for var. *burnettii*. The DEAH helicase unique to H119p4 is one of three DEAH found in the H119p4 genome and was severely truncated and likely inactive. The two full copies of DEAH helicases are present in strains of all varieties.

**Table 2:** (A) Comparison of the three homokaryons using MUMmer.

A: Unique genes in pairwise comparisons between strains		B: Proteins unique to *burnettii* and the position of their genes	
Comparison	# Unique genes		# Proteins
Unique in H39 compared to H119p4 and H97	62	**#Unique to burnettii**	236
Unique in H97 compared to H119p4 and H39	91	Unknown function	223
Unique in H39 compared to H97	100	RecQ DNA helicases	19
Unique in H97 compared to H39	137		
Unique in H119p4 compared to H39 and H97	236	**Position**	
Unique in H97 compared to 119p4	259	Sub-telomeric	78
Unique in H119p4 compared to H97	274	Centromeric	10
Unique in H39 compared to 119p4	281	Other	148
Unique in H119p4 compared to H39	292		

All strains were compared pairwise to each other to identify genomic regions unique to a single strain or present in two strains but absent in the third strain. All differences represented by transposable elements were removed. (B) Genes unique to var. *burnettii* (absent in var. *bisporus*). Remarkable is the considerable number that is located at chromosome ends. Nineteen genes are identified as RecQ DNA helicases. Identical differences were found between var. *bisporus* and var. *burnettii* strains for which Illumina reads were available.

### Recombination landscape of *A. bisporus* var. *burnettii*

**Figure 3:**
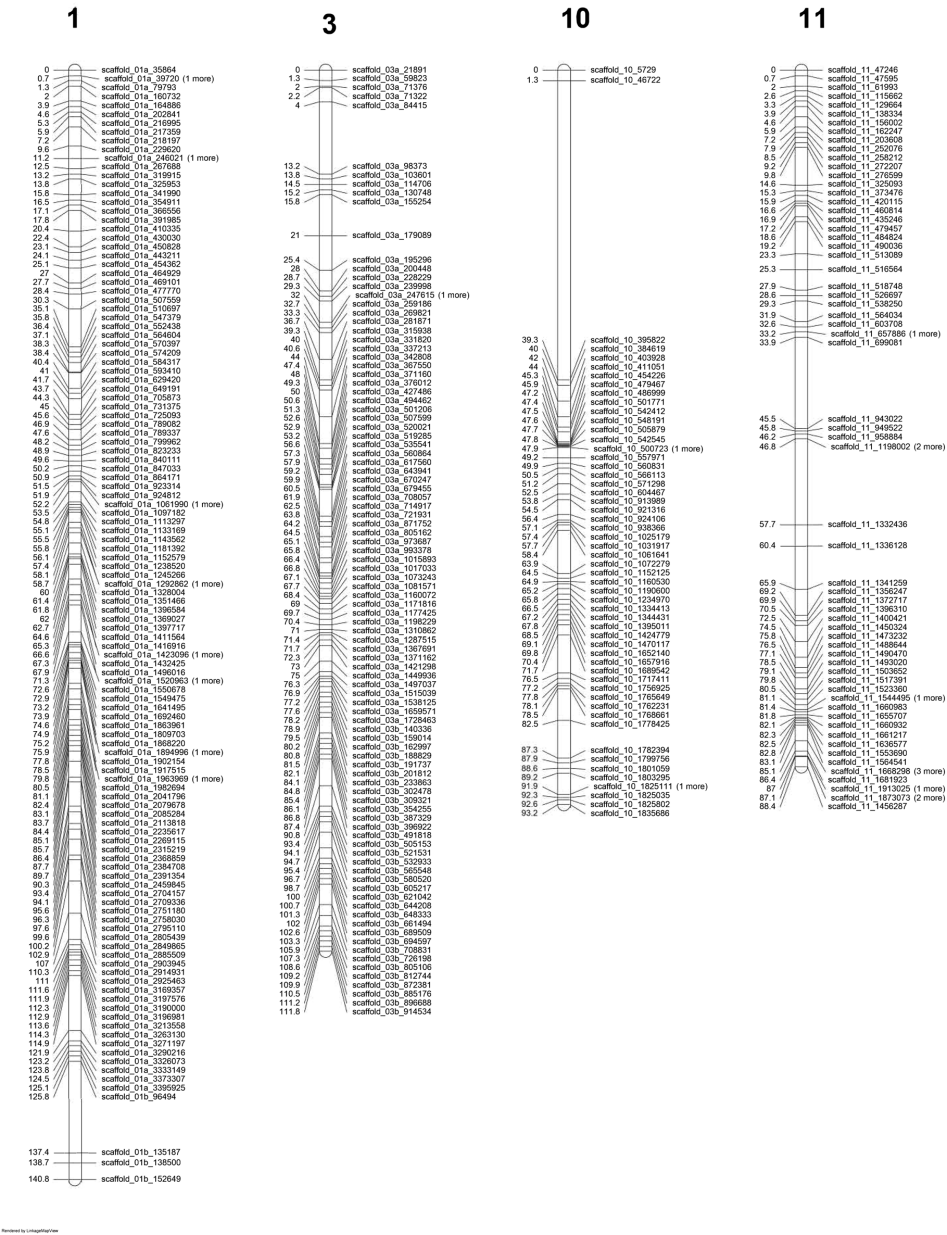
Genetic linkage map of a 119/9 population. Four linkage groups, 1 (consists of two scaffolds), 3 (consists of two scaffolds), 10 and 11 are presented. The SNP markers were labelled as scaffold number followed by their physical positions on that particular chromosome at the right side of each chromosome. The Centimorgan (cM) distance is given per marker pair at the left side of each chromosome. The position of the mating type locus is located as a black bar next to chromosome 1. The map was drawn using R 2.5.1

**Figure 4:**
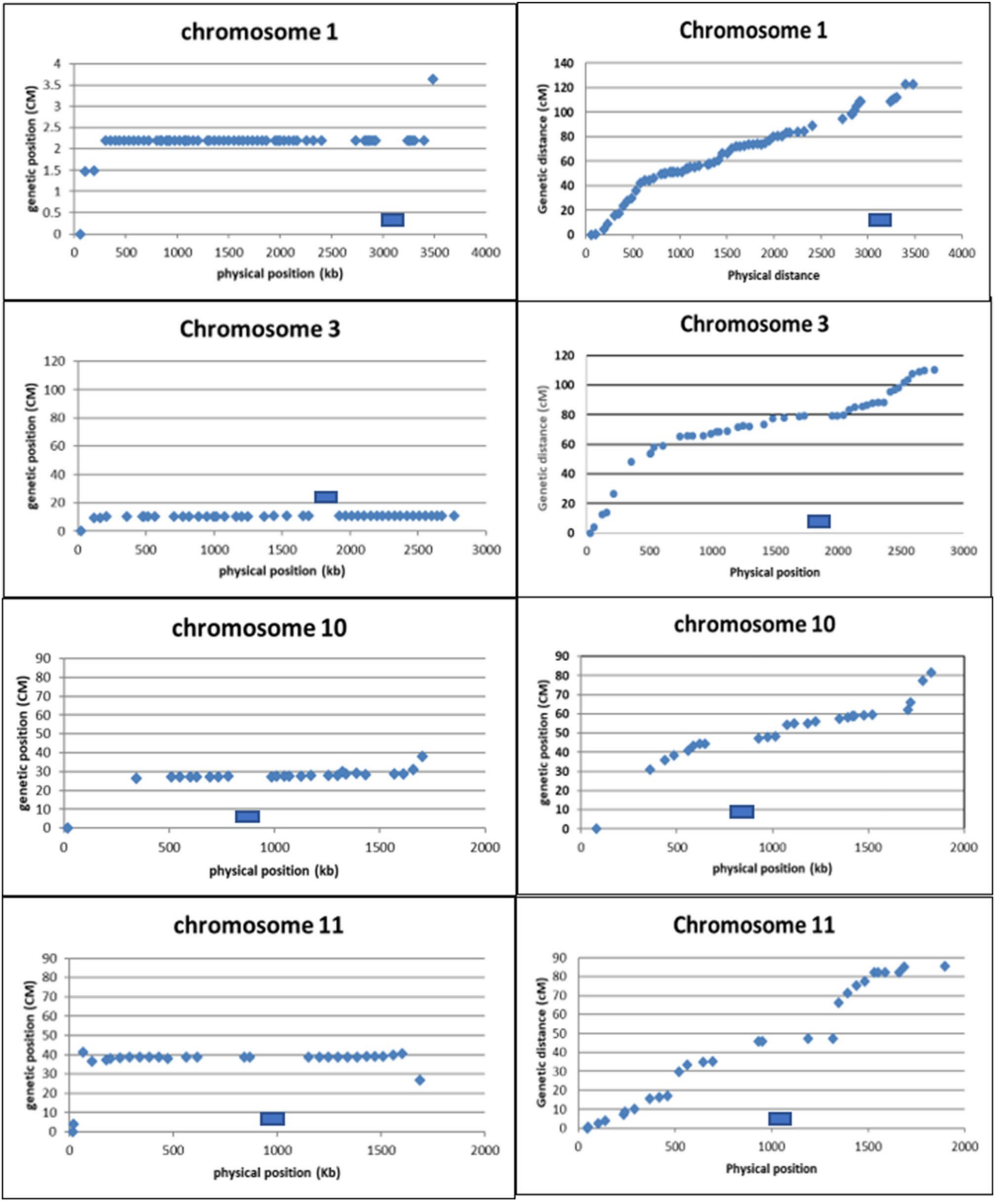
Plots of mapping position (X-axis) of markers against the physical position (Y-axis) for chromosomes 1, 3, 10 and 11 in populations derived from a var. *bisporus* offspring (left column) and the var. *burnettii* offspring (heterokaryon 119; right column). The putative location of the centromere (LRC) on each chromosome is indicated as a blue bar. The plots were generated using Excel (Microsoft Office 2016).

The sequence SBG reads of 154 homokaryotic single spore cultures (SSI) isolated from the heterokaryon 119/9 were mapped against the 16 scaffolds of the de novo assembly of H119p4 and the subsequent filtering steps resulted in retaining SNPs that met all the specified criteria described in M&M. These SNPs were far from evenly distributed over the whole genome. Many areas had none, or a very low number of SNPs due to absence of polymorphism between the genomes of H119p1 and H119p4 (Fig. S3). Chromosomes 2, 5, 7, 9 and 12 are examples of those with hardly any SNPs. Good resolution linkage maps could be made for scaffolds 1 (1a and 1b), 3 (3a and 3b), 10 and 11. After removing co-segregating SNP markers, a total of 354 SNP markers were mapped on these four chromosomes covering ~ 416 cM (Fig. 3). The linkage map clearly indicates a more or less even distribution of recombination over the chromosomes. Plotting the genetic distance of the markers against the physical position for the four chromosomes shows a contrasting recombination landscape between the var. *bisporus *^8^ and the var. *burnettii* (Fig. 4). The var. *bisporus* strain shows only recombination at chromosome ends. The var. *burnettii* strain shows recombination over the entire chromosome, although recombination at chromosome ends is higher (for chromosomes 1, 3, and 10) than for the middle part of the chromosomes. The putative centromeres (see paragraph 2.4) are also indicated in the plots (Fig. 4) and are located in regions where obviously no SNP markers could be generated due to the presence of repetitive elements. For this reason it is not clear if crossovers are possibly suppressed in these regions.

### Large repeat clusters

**Figure 5:**
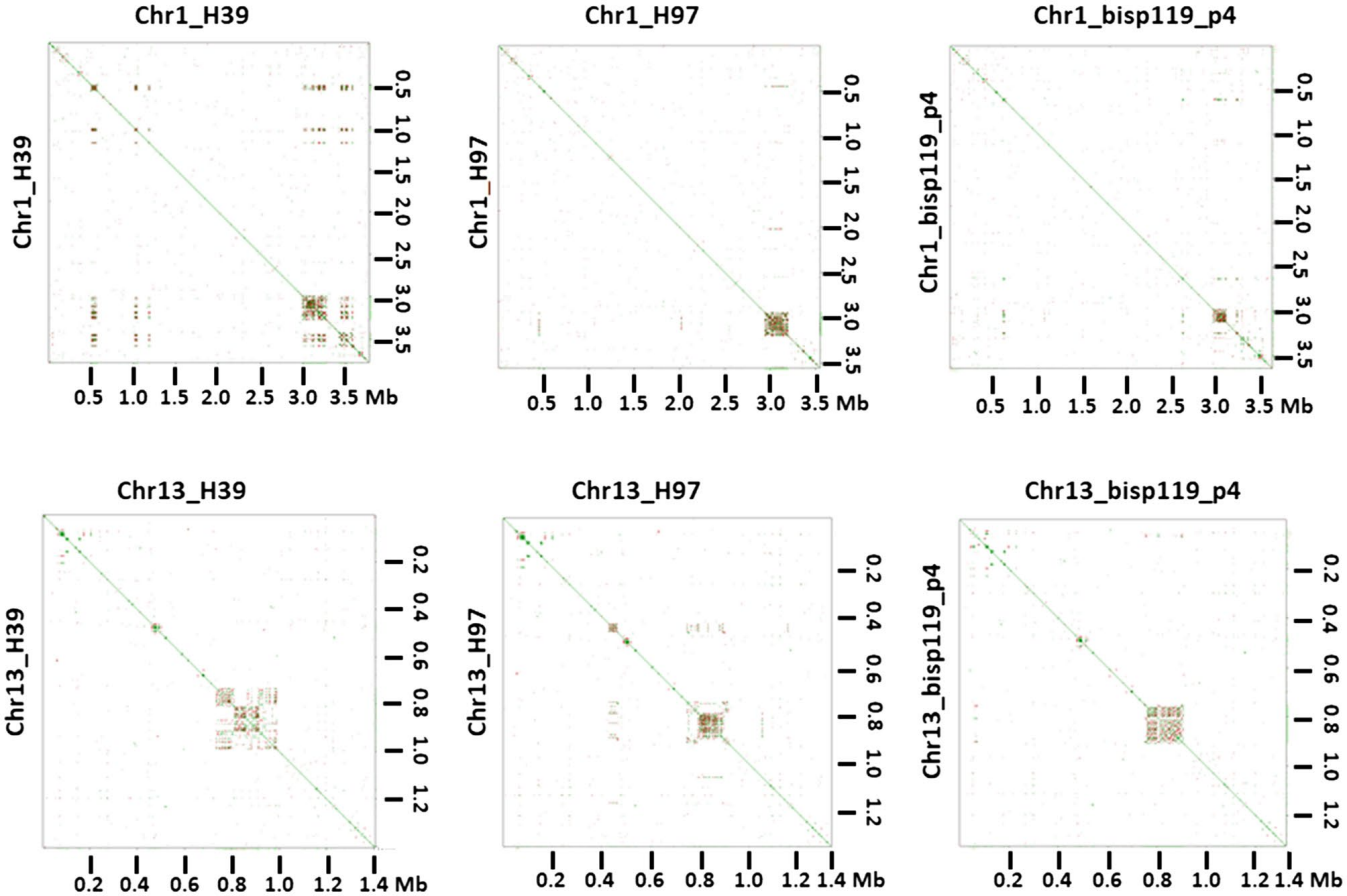
Mummer plots of chromosome 1 and 13 of homokaryons H39, H97 and H119_p4. Chromosomes were plotted against themselves using D-Genius, revealing clusters of repetitive elements in each chromosome. The plots clearly show that each homologous chromosome has a large repeat cluster (LRC) on an almost identical position, likely representing the centromere. The figure was generated with MAFFT version 7 (https://mafft.cbrc.jp/alignment/server/large.html).

Mummer plots of chromosomes plotted against themselves revealed that every chromosome has one large repeat cluster (LRC). The position of this cluster varies between the different chromosomes within a strain but is located in nearly the same position on each homologous chromosome of the three strains (Figs. 5, S1). Contrastingly, smaller repeat clusters are not always located on identical positions between strains. The LRC vary in length between chromosomes within a strain and between the homologous chromosome of all three strains with lengths varying between 110 and 280 kb.

Although genome analysis for repetitive elements has been described previously for the genomes of *A. bisporus*^10,12^, a thorough identification and annotation has not been done. Here we annotated the LRCs of all chromosomes, of each strain, as described in M&M. A library was built of prototypes of genomic sequences of each transposable element, and of proteins for each transposable element if sufficient codon sequences could be found. In total, 202 families were identified distributed over Class I (retrotransposons) and Class II (DNA transposons) (Table 3). In addition, 22 repetitive elements were found that could not be classified (designated as Unknowns). Full length copies of each family were Blasted against the genome of each strain and used to annotate each genome for all repeat families. A comparison with RepeatMask of homokaryon H97 (not shown) showed that the manual annotation done here is clearly more complete (except for mini- and microsatellites). A list of the presence or absence of the elements in each strain is given in supplemental data (Table S2). For each family of transposable elements, the specificity for the LRC was determined: (type 1) LRC-specific (present only in LRCs and in all LRCs); (type 2) present in all LRCs, but not exclusive in LRCs, (type 3) exclusive in LRCs but not in all, (type 4) non- specific. One family of Long Terminal Repeat (LTR)-retrotransposons, i.e. Copia-28, four families of non-LTR-retrotransposons (HaTad-type) and two Unknown Repeats were present exclusively and in all LRCs (type 1) in all strains. Only for the two Unknow Repeats a few small fragments are also seen outside the LRC. Next to these exclusive elements, two Gypsy families were present in all LRCs but also found outside these clusters (type 2). Finally, a number of elements were exclusively found in LRC but only in one or a few (type 3, Table S2). For most of the retrotransposons, at least one full length copy was found in the genome with ORF’s coding for all conserved motifs known for the specific type of retrotransposon. The majority of the retrotransposon elements in the LRC represent truncated copies, often caused by jumping within the same type or other families of retrotransposons.

**Table 3:** Overview of transposable elements annotated for the genomes of homokaryons H39, H97 and H119p4.

Class I (retrotransposons)	Type	# Families
LTR-retrotransposons	Copia	26
	Gypsy	21
DIR	Ngaro	10
Non-LTR retrotransposons	L1	10
	HaTad	5
	Tad	5
Penelope-like elements	Penelope	3
**Class II (DNA transposons)**		
En/Spm		20
Dada		15
hAT transposase		7
	Abr1	1
KDZ transposases	Kyakuja	4
	Zisupton	14
Plavaka_transposase		28
Mariner_TC		3
Helitron		22
MITE		8
Total # families		202
Unknown repeats		22

The Class I, Class II and the Unknown elements in the LRC represent 79 to 85% of the LRC-sequences and of these, the majority were Class I retrotransposons (Table 4). Although a large number of different transposable elements are found in each LRC, the main part of the LRC sequence is covered by the LRC-specific repeats (type 1), i.e. 46–53%.

**Table 4:** Statistics of the annotation of LRCs.

% of LRC annotated with repeats		% of LRC annotation			% LRC-specific (type 1)
Homokaryon		Class_I (%)	Class_II (%)	Unknown (%)	
H39	85%	80	8	12	46
H97	79%	78	10	12	45
H119_p4	84%	85	5	10	53

Between 79 and 85% of the LRCs are covered by TE and unknown repeats. The majority of the repeats are formed by retrotransposons (Class I). A substantial part of the annotated repeats are LRC-specific (type 1).

Despite sharing a number of transposable elements, alignments of LRCs of different chromosomes and homologs of the three strains show that there is no collinearity and LRCs differ thus considerably in positions and number of transposable elements (Fig. S4). Exceptions are the LRCs of chromosomes 5, 6, 9 and 10 of homokaryons H39 and H97 which show a high degree of collinearity (Fig. S5). This indicates that both strains share a common ancestor, which is supported by the fact that in the genomic regions containing collinear LRCs the SNP frequency between H39 and H97 is very low (Fig. S6).

### Methylation

**Figure 6:**
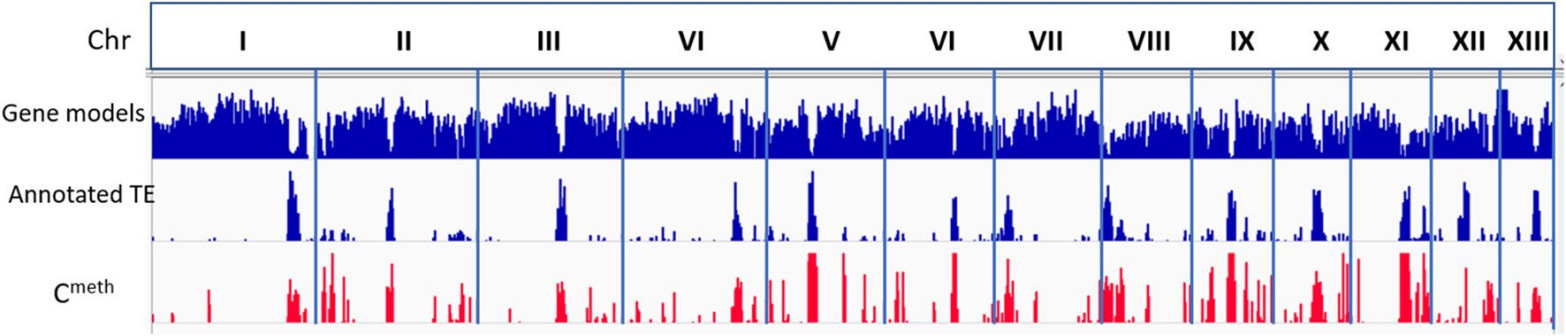
Genomic profiles of gene models, annotated transposable elements (TE) and CpG methylation (C^meth^) of the 13 chromosomes (Chr) of homokaryon H97. Each chromosome has a clear large repeat cluster shown as the largest annotated TE peak in each chromosome. The pattern of the annotated repeats coincides well with the CpG methylation profile. The figure was generated using Integrative Genomics Viewer version 2.5.2.

The genomes of the constituent nuclei of Sylvan A15 are both collinear with, and highly similar to that of homokaryon H97^8^. The bisulphite treated reads, previously generated for Sylvan A15, could thus be mapped well on the genome of H97 and could be applied to visualize methylation of genomic DNA in this homokaryon. The methylation pattern (CpG methylation) nearly perfectly matches with the annotated repeats in the genome of homokaryon H97, indicating that methylation is almost completely restricted to transposable elements (Fig. 6). Visual inspection of the LRCs showed that all LRCs are heavily methylated and for each chromosome of H97 the LRC is the largest and most densely methylated region.

## Discussion

Many fungal WGS’s are available now, including those of basidiomycetes. They all have in common, however, that the number of scaffolds far exceeds the number of chromosomes, the annotation of repeats is incomplete or incorrect (especially centromeres) and for most species only for one strain a WGS is available. Recently, whole genome sequences of two additional homokaryons of *A. bisporus* var. *bisporus* have been published, both having incompletely annotated repetitive elements, or a number of scaffolds that far exceeds the number of chromosomes^13,14^. One of the problematic issues in reconstructing complete genomes has been the reliable sequencing of long stretches of repetitive DNA which has now been significantly improved by long-reads sequencing technologies such as PacBio.

Here we present a de novo sequenced homokaryon of *A. bisporus* var. *burnettii* and an update of the previously de novo sequenced homokaryons of *A. bisporus* var. *bisporus*. For two homokaryons (H39 and H119p4), three chromosomes each consist of two scaffolds. Alignment of homologs of these chromosomes between the three homokaryons indicates that the gap between the two scaffolds of these chromosomes is likely to be small. The exception is the gap between the two scaffolds of chromosome 5 of H119p4. Here a gap is estimated to be ca 210 kb representing sequences present in H39 and H97 but absent in H119p4. Screening of the Illumina reads of H119p4, however, showed that these sequences are present in H119p4 as well, and were apparently missed in the assembly. Three of the six gaps coincide with a large repeat cluster, again indicating that occasionally even long-reads sequencing techniques can have problems with long stretches of highly repetitive DNA. In addition to these gaps, six telomers are missing in the genome sequences of the homokaryons (1 for H39, 2 for H97 and 3 for H119p4). For two telomere-missing chromosome ends in H119p4 (chromosome 3 and 12), Penelope like retrotransposons were identified. Since these elements are closely linked to telomeres in many organisms^15^, including *A. bisporus*^8^, this indicates that these chromosomes are nearly complete. Remarkable is the missing of the telomere sequence at the right end of all chromosome 9 homologs where the rDNA cluster is located. It is possible that this type of repeat hampers the complete sequencing of chromosome 9 homologs. In other fungi, a telomer sequence was found next to the rDNA cluster^16,17^ which might indicate that chromosome 9 is missing a telomere but is nearly complete.

Centromeres in diverse organisms, including fungi, can be divided in point centromeres and regional centromeres. The former usually represent a short and unique DNA sequence which supports a kinetochore with only one microtubule attached^18^. Regional centromeres span a large segment of a chromosome and support multiple microtubule attachments^19^. Such centromeres often contain intact and/or truncated transposable elements^20^. The presence of a LRC with variable lengths of 110 to 280 kb on each chromosome suggests that these represent regional type centromeres. This is supported by the fact that the LRCs are located in the same position in all chromosome homologs (but vary in position between non homologous chromosomes), are heavily methylated and consist mainly of transposable elements. A conservation of the position of centromeres has been found in ascomycetes in genetically unrelated *Candida albicans* strains^21^ and in three strains of *Neurospora crassa*^22^. The only basidiomycetes for which centromeres have been annotated thus far are the non-mushroom forming *Cryptococcus neoformans* and *Microbotrium lychnidis-dioicae*^20^, both having regional centromeres of 5–10 kb. The putative centromeres of *A. bisporus* are much larger and resemble in length to those of *N. crassa*^23^. LRC flanking regions align well for all homologous chromosomes of the three homokaryons indicating that only the LRCs vary in sequence (Fig. S7). In many organisms also a methylation of the pericentromeric region has been observed^24^. In *A. bisporus* we see, however, a clear border between the methylation in the LRC and the absence of methylation in the flanking regions (Fig. S7). As stated above, all LRC nearly completely consist of repetitive elements. A few putative gene models that did not represent repetitive elements where found in LRCs in each strain, i.e. 36, 54 and 74 genes for H119p4, H39 and H97, respectively (Table S3). These are all small genes (average genomic length < 1 kb) and for only four genes a putative function could be found (Table S3). In general, the annotation of repetitive DNA is done with efficient but error-prone automated processes. A philosophy behind one of the best databases for repetitive elements, i.e. Repbase, has therefore been to incorporate a significant amount of manual curation into the database^25^. We have, therefore, annotated the large repeat cluster present on every chromosome in the three homokaryons mainly manually, a laborious process but with an accurate result. Such a complete annotation of putative centromeres of all chromosomes in genetically unrelated strains of a basidiomycete has not been done previously. It appeared that especially in the LRC the majority of the elements are truncated by jumping of elements within the same or into other elements which makes it impossible to have a correct automated annotation. A comparison between Repeat Mask identification of repetitive elements in the H97 genome and the manual annotation showed that the latter is clearly more complete. A comparison of the manually annotated elements with the repetitive elements of *A. bisporus* present in the NCBI database shows that these were all covered by the new annotation. Nearly 50% of repetitive elements are specific to the LRCs and are heavily methylated. This supports the general opinion that the recognition of centromere sequences by kinetochore proteins is a mixture of genetic and epigenetic factors^20^. The AT content of the LRCs is identical to that of the overall genome indicating that the transposons in *A. bisporus* are not degenerated by a mechanism known as “repeat-induced point mutation” (RIP) as seen in *N. crassa*^26^. Only chromosomes 5, 6, 9 and 10 of homokaryon H39 and H97 have nearly collinear LRCs, which indicates a shared ancestor. Previously, we have generated SNP data for the genomes of H39 and H97 using Illumina data^8^. Plots of the SNP frequencies for these chromosomes show that these LRCs are positioned in a region with a very low SNP density (Fig. S6), indeed an indication of a shared ancestor. The SNP density in the LRCs is somewhat higher indicating that the LRC regions have a larger mutation rate than other parts of the genome. This is supported by a comparison between one of the retrotransposons (Gypsy-1_AB) that occur inside and outside centromeres for homokaryon H97, and that showed a 4.6% deviation in sequence from the consensus sequence for the 19 copies inside the putative centromeres while 1.7% deviation is found for the 51 Gypsy copies outside the centromeres (Table S4). The reason for this difference in mutation rate is unknown but a higher mutation rate in centromeres than in other genomic regions has also been observed in yeast^27^.

The linkage map of four (out of 13) chromosomes generated by the offspring of the heterokaryon 119/9 clearly indicates a more even distribution of crossovers over the chromosomes than in var. *bisporus*. Although only four chromosomes were covered, it is reasonable to assume that other chromosomes will have a similar recombination landscape. The absence of SNPs in many genomic regions limited the linkage map to only four chromosomes and this might indicate that the heterokaryon 119/9 originates from a cross between two compatible single spore isolates from the same spore print. The 119/9 heterokaryon originates from the ARP collection^11^ and the *burnettii* strains were distributed as fragments of lamellae of wild isolates. It is quite possible that during the isolation of single spore isolates from these lamellae, mating has occurred between siblings. Previously, the locus linked to the number of spores per basidia (BSN) has been located on chromosome 1 in or near the LRC of chromosome 1. After filtering out repetitive elements, we have compared putative genes within the LRC on chromosome 1 and the left flanking area, the putative region where BSN is located. No difference could be found between the *bisporus* homokaryon H97 and the *burnettii* homokaryon H119p4 other than small fragments that have no known function. The genetic base for the differences in basidial spore numbers remains thus unknown. The comparison between the gene models of the three homokaryons identified 236 proteins that are unique to the *burnettii* strain H119p4. A large part of these genes (76) are located in subtelomeric regions. Only 19 have a hit with known genes and represent RecQ like helicases. Helicases use ATP to bind, unwind, or remodel RNA or DNA in many facets of nucleic acid metabolism. They contain a conserved ‘helicase core’ of two RecA-like domains but act on varied substrates by different mechanisms^28^. Helicases are divided in superfamily SF1 and SF2^29^. All families of these two superfamilies identified in yeast are also present in both varieties of *A. bisporus* (Table S5). The exception are the RecQ helicases found in subtelomeric regions in all var. *burnettii* strains which are absent in the var. *bisporus* strains. They resemble the RecQ helicases also found in subtelomeric regions of a number of other fungi^30–32^ but are absent in many fungal WGS’s, possibly due to difficulties in sequencing chromosome ends. The subtelomeric RecQ helicases are quite different from non-subtelomeric RecQ helicases for which structural and functional analyses have been done^28^. The latter represent motor proteins with 3′–5′ DNA helicase activity and unwind DNA in an ATP-dependent manner, requiring Mg2+ as a cofactor^33^. Next to the helicase core sequence with its conserved motifs, most RecQ helicase also contain a Zinc binding domain, a RecQ C-terminal (RQC) domain believed to mediate protein–protein interaction, a helicase and RNase D C-terminal (HRDC) domain involved in interactions with nucleic acids^29^. These RecQ helicases have been found in bacteria, fungi, animals, and plants, and their copy number ranges from one in *Escherichia coli* and *Saccharomyces cerevisiae* to up to seven in *Arabidopsis*^28^. Both varieties of *A. bisporus* have three copies that resembles these non-subtelomeric RecQ helicases. Only one of these has the additional domain structures next to the helicase core domain. In none of the subtelomeric RecQ helicases of *A. bisporus* var. *burnettii* or other fungi, domains other than the helicase core could be found (Fig. 2SA). A Maximum likelihood comparison of the helicase core domain of all RecQ helicase genes also shows that the subtelomeric RecQ helicases found in var. *burnettii* form a separate group of helicases (Fig. S2B). It has been suggested that the subtelomeric position of these RecQ helicases indicate their involvement of telomere maintenance^34^. The absence of these type of helicases in the var. *bisporus*, however, does not seem to lead to any (telomeric) abnormality indicating that the subtelomeric RecQ helicases might play another role. The helicase core domain of the subtelomeric RecQ helicases all have the typical motifs characteristic for helicases^35^ (Fig. S8) and are thus likely active. RecQ helicases play a role in meiotic recombination but although tempting to speculate, it is unclear if they have any role in the difference in the meiotic recombination landscape of var. *burnettii* and var. *bisporus* strains.

The availability of high quality whole genome sequences and the contrasting recombination landscape of the two subspecies of *A. bisporus* offers the opportunity to do segregation analysis for the recombination landscape and find the genetic components determining positions of crossovers. That is not only very important for breeding of the button mushroom, but might also be relevant for breeding in other crops such as plants. Knowledge of mechanisms determining positions of crossovers might also offer opportunities to direct recombination to regions of interest or avoid recombination of genomic areas were the maintenance of allelic combinations is wanted. In addition to studying differences in life cycles, the button mushroom could thus be a model organism for studying an important element of meiosis, i.e. a mechanism involved in positioning of crossovers.

## Materials and methods

### Sequencing

High molecular DNA isolation and PacBio RSII sequencing of homokaryon H119p4 was done as described previously^8^. The constituent homokaryons of heterokaryon 119/9, i.e. H119p1 and H119p4 were paired-end sequenced with Illumina HiSseq platform. Due to the high coverage of PacBio RSII reads of the H119p4 genome, the HiSseq reads were not needed to complete the H119p4 genome.

### De novo assembly

The PacBio RSII reads were assembled using the SMRTportal v2.2.0 HGAP2 algorithm^36^ and the assembly was polished using the SMRTportal 2.3.0 Quiver algorithm (PacBio, Rockville, MD). To connect contigs into scaffolds, SMRT View BridgeMapper (https://github.com/PacificBiosciences/Bioinformatics-Training/wiki/Bridgemapper) was used in combination with a genetic linkage map based on SBG data (“Sequenced based genotyping” section). Ends of incomplete scaffolds were BLASTed (CLC Genomics Workbench version 7.5.1, Qiagen, Aarhus, Denmark) against the full de novo assembly to find evidence for connecting incomplete scaffolds.

### Sequenced based genotyping

As a segregating population, 154 homokaryotic single spore cultures (SSI) were isolated from the heterokaryon 119/9. Together with the parental homokaryons H119p1 and H119p4, these were genotyped using Sequenced Based Genotyping (SBG) (Keygene N.V. owns patents and patent applications protecting its Sequence Based Genotyping technologies) as previously described^37,38^. ApeKI was used as a restriction enzyme. The raw SBG Illumina paired-end sequencing data was demultiplexed based on their barcodes and for each barcode, the number of reads were counted. The number of reads per barcode ranged between 500,000 and 22,000,000. To select SNPs for the generation of a linkage map, all sequencing reads were aligned against the de novo assembly of *A. bisporus* H119p4 as a reference, using the Burrows-Wheeler Alignment tool (BWA) backtrack algorithm of the BWA version 0.7.7-r441^39^. The generated alignment files were used as input for variant calling using FreeBayes version v0.9.18-g4233a23 with default settings^40^. The resulting VCF output file was initially filtered for a per sample read depth > 5 and the loci of type “SNP”, retaining 605,198 valid SNP loci. These SNP loci were filtered once more using a previously published filtering pipeline^41^ to obtain enough loci (8,225) and individuals (154) to construct a genetic linkage map. The SNP loci of scaffolds 1 (1,436 loci), 3 (1,295 loci), 10 (718 loci) and 11 (780 loci) were extracted from the dataset to be used as described in “Linkage map” section.

### Linkage map

Due to the large amount of SBG data, the DOS version of RECORD^42^ was used to order the loci and identify all identical loci taking into account missing alleles. For each group of identical loci, one locus was selected to represent the entire group, resulting in a dataset of 2,504 loci for the four scaffolds. These unique loci were used as an input for JoinMap 4.1 software to construct the genetic linkage map of 119/9 based on the independence LOD score using a haploid model (HAP1) with default parameter settings (independence LOD score; significance level from 2.0 to 10.0 LOD^43^). Chi-square analysis (P < 0.05) was used to check the accuracy of fitting to the expected Mendelian ratios and linkage groups (LGs) were set up at the level of LOD ≥ 5. Centimorgan (cM) distances were expressed by the Kosambi function^44^ and crossover frequency was calculated as the crossover number per individual per chromosome. We plotted the number of map units of SNP markers per linkage group calculated with JoinMap 4.1 against their respective physical distance (bp) to detect the relationship between genetic and physical distance.

### Gene prediction

Gene prediction was performed using BRAKER^45^, which is an algorithm that self-trains using RNA-Seq expression evidence and produces parameter files that can be used by the gene predictor AUGUSTUS^46,47^. We used an iterative approach to incrementally improve the quality of gene predictions based on RNA-Seq evidence, as outlined in this section.

First pooled RNA-Seq reads from a previously published genome-wide transcriptomics analysis during development (NCBI accession PRJNA309475^48^) were aligned to the assembly of each strain using Hisat version 2.1.0^49^ with a minimum intron length of 20 bp and a maximum intron length of 1,000 bp. Next, BRAKER version 2 was used to train (i.e. generate parameter files for) the gene predictor AUGUSTUS version 3 on the assembly of strain H97. These parameters files were used to predict genes in all three strains, in combination with assembly-specific intron hints generated by BRAKER based on the RNA-Seq alignments. Manual inspection of the initial gene predictions revealed that a considerable number of genes contained introns with poor RNA-Seq support. To address this, the set of intron hints that were generated by BRAKER was trimmed by only retaining introns with support of at least 15 RNA-Seq reads. AUGUSTUS was run with these high-quality intron hints and the previously generated BRAKER parameter files (gene set 1). Manual inspection revealed that a considerable number of chimeric genes were predicted, i.e. neighbouring genes that were erroneously merged into one gene model by a relatively large and unsupported intron. To address this, we identified predicted introns that were longer than 150 bp and unsupported by RNA-Seq reads. The midpoints of these introns were labelled as ‘intergenic’ hints (forcing AUGUSTUS to break up the chimeric gene). These hints were combined with the previously generated intron hints and supplied to the gene predictor AUGUSTUS to generate a new set of gene predictions (gene set 2). In parallel, genes were predicted with AUGUSTUS without using hints (gene set 3). Finally, the gene sets were merged in the following order: if a gene in set 1 had no overlap with any gene in set 2 then this gene was added to set 2, and subsequently if a gene in set 3 had no overlap with any gene in set 2 then this gene was added to set 2. The result is a set of non-overlapping genes for each strain.

An effort was made to synchronize the gene names between the strains. Gene names of H97 were (conservatively) mapped to strains H39 and H119p4 if there was a clear ortholog. Here, ‘ortholog’ is defined as a bidirectional best blastp hit (reciprocal best hit) with a minimum E-value of 1e−10 and a reciprocal sequence coverage of 90% (which means that the orthologs should be very similar in both sequence and length). Furthermore, the genetic context of the orthologs should be largely syntenic, which means that at least 50% of the neighbouring genes of the ortholog in strain H97 should correspond to the neighbouring genes of the ortholog in the other strain. If an ortholog was found using these (conservative) criteria, then the gene received the same protein ID as in strain H97. If a gene did not have an ortholog, then it was given a protein ID that is unique in all three strains.

### Functional annotation of the predicted genes

The functional annotation of the predicted genes/proteins was performed essentially as previously described^50^. Conserved protein domains were predicted using PFAM version 32^51^ and were subsequently mapped to their corresponding gene ontology (GO) terms^52,53^. Proteases were predicted using the MEROPS database^54^ with a BlastP E-value cutoff of 1e−5. Secretion signals were predicted using Signalp 4.1^55^. Transmembrane domains were predicted using TMHMM 2.0c^56^. Proteins with a secretion signal, no transmembrane domain (except in the first 40 amino acids) and a total length shorter than 300 amino acids are considered small secreted proteins. Genes and gene clusters involved in secondary metabolism were predicted using a pipeline based on the SMURF method^57^. SMURF parameter d (maximum intergenic distance in base pairs) was set at 3,000 bp, and SMURF parameter y (the maximum number of non-secondary metabolism genes upstream or downstream of the backbone gene) was set at 6. BUSCO version 2.0 (database fungi_odb9) and CEGMA were used to determine the completeness of the assembly and gene predictions^58,59^. For BUSCO, the completeness score was defined as the sum of the reported percentages of ‘complete’ and ‘fragmented’. The gene predictions and functional annotation can be browsed interactively on https://fungalgenomics.science.uu.nl/.

### Comparative genomics of the three strains

Orthofinder version 1.0.7^60^ was used to group the predicted proteins into clusters based on homology. In this context, these clusters can be interpreted as protein families. An inflation parameter of 1.5 was used during the MCL clustering step. In a separate approach, NUCmer (part of the MUMmer software package version 3.23) was used for pairwise comparisons of the assemblies of the strains^61^ using the ‘–mum’ parameter. For each strain, unique regions were identified in pairwise comparisons with the two other strains individually, as well as with both other strains simultaneously. Initial results showed that repeat-associated regions formed a large part of the identified regions, and for this reason we decided to use the previously identified repetitive regions (see above) for masking. Specifically, each previously identified repetitive region was extended on both ends with 1 kb of sequence, and subsequently neighboring repetitive regions (less than 1 kb of sequence apart) were merged. A unique region was reported if its length was at least 2.5 kb, as well as any genes that overlapped with this region for at least 50% of the gene’s length. The genes that were identified in this manner were inspected manually and transposon-associated genes were removed from the set.

### Annotation repetitive elements

Considering the complexity of the large repeat clusters (LRC), the annotation was done mainly manually. Each LRC was Blasted for repetitive elements against Repbase^62^. Each element found was Blasted against the whole genome of all three strains to find the complete or largest sequence for this element. Orf’s were identified and Blasted to the NCBI database to annotate each repeat. The full length (or largest) DNA copy was Blasted against the genome of each strain to generate gff files for annotation of each element for the whole genome of all three strains. A library was generated of genomic prototypes of each family and ORFs, if complete sequences were available. Repetitive elements were considered as a separate family when they shared less than 80% genomic similarity to any other element. The library was checked for the NCBI deposited proteins of transposable elements of *A. bisporus* which showed that all NCBI sequences were present in the created library. Sequences with no hit in Repbase or NCBI but representing a repetitive element in the genomes were annotated as Unknowns. The library was submitted to RepBase. The overlap between the repetitive elements and the gene predictions were calculated using the Jaccard statistic as implemented in Bedtools (version 2.29.2).

### Methylation analysis

Methylation data of the variety Sylvan A15, done in a previous (unpublished) experiment, was used here to examine methylation of the genome of H97. Previously, we have shown that Sylvan A15 was a second generation derivative of the variety Horst U1 by isolation of a heterokaryotic single spore culture^8^. Horst U1 is a hybrid generated by crossing the homokaryons H39 and H97. Due to the pairing of non-sister nuclei after meiosis and the restriction of recombination to extreme ends of chromosomes, genomes of such fertile single spore cultures are nearly identical to the parent^8^. The reads of bisulphite threated DNA of A15 can thus be mapped well to the genome of H97 and used for DNA methylation analysis in H97.

Vegetative mycelium of a commercial variety (Sylvan A15) was bisulphite treated (ServiceXs, Leiden. The Netherlands) and sequenced (Illumina HiSeq 2000). The main C-methylation was found in CpG (6.6%) whereas CHC and CHH was found in 0.7 and 1.1% respectively. For the analysis only the CpG data were used. After filtering, the reads were mapped against the genome of H97.

## Supplementary information

Supplementary Information 1.

Supplementary Information 2.
