# Author Correction: Telomere-to-telomere assembled and centromere annotated genomes of the two main subspecies of the button mushroom *Agaricus bisporus* reveal especially polymorphic chromosome ends

**DOI:** 10.1038/s41598-021-96123-y

**Published:** 2021-08-12

**Authors:** Anton S. M. Sonnenberg, Narges Sedaghat-Telgerd, Brian Lavrijssen, Robin A. Ohm, Patrick M. Hendrickx, Karin Scholtmeijer, Johan J. P. Baars, A. van Peer

**Affiliations:** 1 Plant Breeding, Wageningen University and Research, Droevendaalsesteeg 1, 6708 PB Wageningen, The Netherlands; 2 Ceradis B.V., Agro Business Park 10, 6708 PW Wageningen, The Netherlands; 3 Department of Microbiology, University of Utrecht, Padualaan 8, 3584 CH Utrecht, The Netherlands

Correction to: *Scientific Reports* 10.1038/s41598-020-71043-5, published online 04 September 2020

The original version of this Article contained errors in Figure 4.

The left and right panels for chromosome 1 were incorrectly swapped.

Additionally, in the right hand panel of chromosome 10 and chromosome 11, the y-axis data points “0; 10; 20; 30; 40; 50; 60; 70; 80; 90” were incorrectly given as “0; 20; 40; 60; 80; 100”.

The original Figure 4 and accompanying legend appears below.

**Figure 4:**
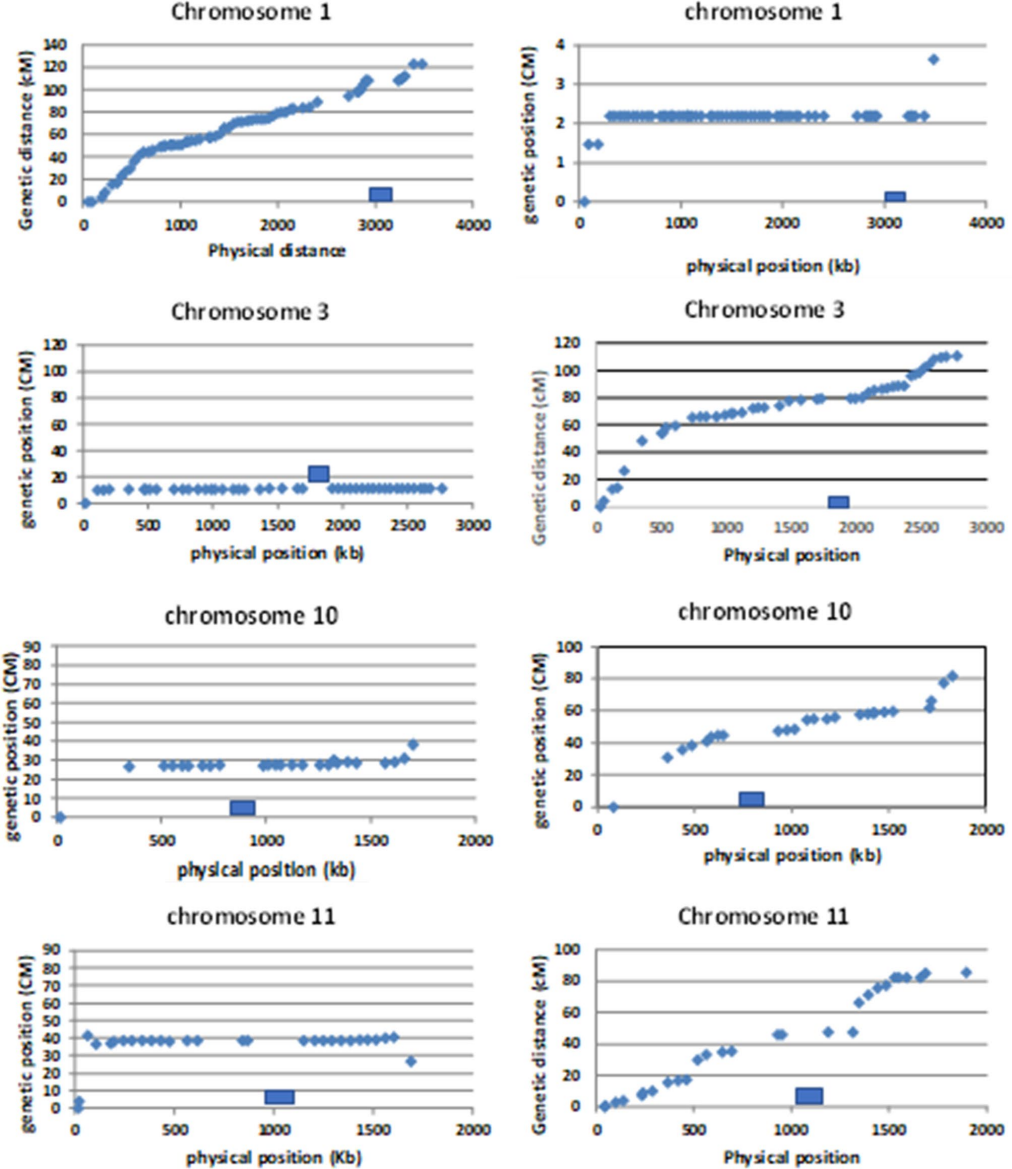
Plots of mapping position (X-axis) of markers against the physical position (Y-axis) for chromosomes 1, 3, 10 and 11 in populations derived from a var. *bisporus* offspring (left column) and the var. *burnettii* offspring (heterokaryon 119; right column). The putative location of the centromere (LRC) on each chromosome is indicated as a blue bar. The plots were generated using Excel (Microsoft Office 2016).

The original Article has been corrected.

