# Supplementary material for: Telomere-to-telomere assembled and centromere annotated genomes of the two main subspecies of the button mushroom Agaricus bisporus reveal especially polymorphic chromosome ends
Source: Sci Rep. 2020 Sep 4;10:14653. doi: 10.1038/s41598-020-71043-5 (PMC7473861; doi:10.1038/s41598-020-71043-5)
Supplement: Supplementary file 1 — Supplementary Information 1. [file 41598_2020_71043_MOESM1_ESM.docx]

Telomere-to-telomere assembled and centromere annotated genomes of the two main subspecies of the button mushroom *Agaricus bisporus* reveals especially polymorphic chromosome ends.

Anton S.M. Sonnenberg^1*^, Narges Sedaghat-Telgerd^2^, Brian Lavrijssen^1^, Robin A. Ohm^3^, Patrick M. Hendrickx^1^, Karin Scholtmeijer^1^, Johan J.P. Baars^1^, A. van Peer^1*^

^1^Plant Breeding Wageningen University and Research, Droevendaalsesteeg 1, 6708 PB, Wageningen, the Netherlands; ^2^ Ceradis B.V., Agro Business Park 10, 6708 PW Wageningen; ^3^ Department of Microbiology, University of Utrecht, Padualaan 8, 3584 CH, Utrecht, the Netherlands.

Figure S1. Mummer plots of chromosomes plotted against themselves reveal the presence of a large repeat cluster (LRC) on each chromosome. The LRC is located at nearly the same position in each chromosome homologue and some chromosomes show additional repeat clusters (especially chromosome 8).

Figure S2. A: Graphic representation of RecQ helicases of the non-subtelomeric type found in both *A. bisporus* varieties and in *Saccharomyces cerevisiae* (first and second line) and RecQ helicases found in subtelomeric regions (bracket) present in only in the var. *burnettii* and an example found in the subtelomeric region of the basidiomycete *Metarhizium anisopliae* ^1^.

B: Maximum Likelihood tree of the helicase domain of RecQ helicases (non-subtelomeric and subtelomeric) of *A. bisporus* var. *burnettii* and some other fungi. The subtelomeric group (bracket) is clearly separated from the non-subtelomeric RecQ helicases. For var. *burnettii* only one gene is included of the group of 10 identical subtelomeric RecQ helicases. For other fungi the genebank number is indicated after the species name. The tree is drawn to scale, with branch lengths measured in the number of substitutions per site. The analysis were conducted in MEGA X ^2^

Figure S3. SNP frequency distribution between H119p1 and H119p4. Many chromosomes of the 2 constituent heterokaryons of 119 have large stretches of low SNP frequencies indicating homology.


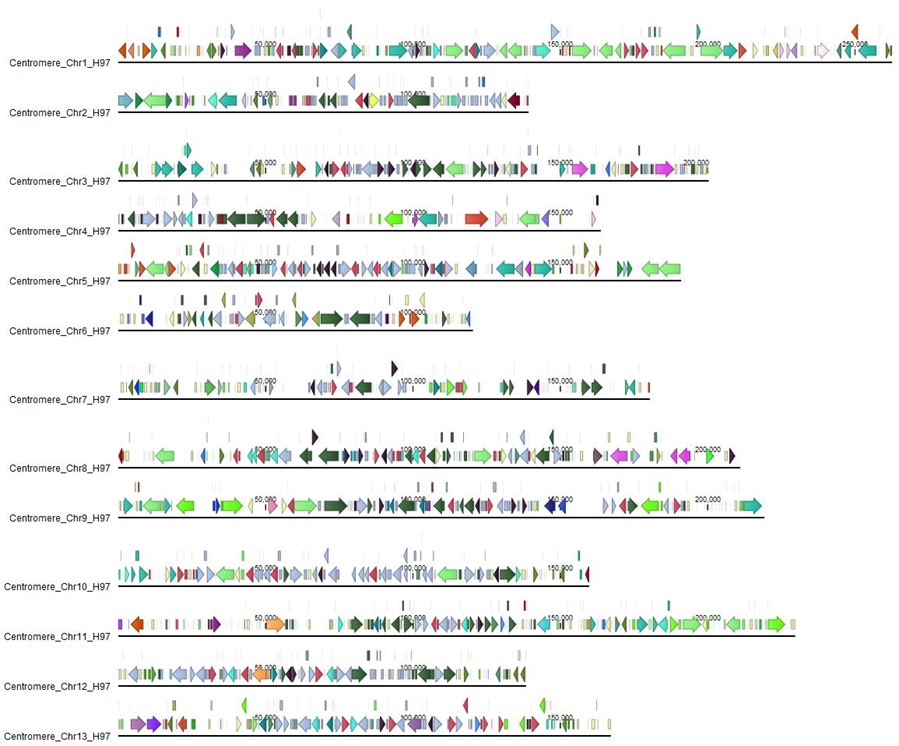


Figure S4. Annotated Large Repeat Clusters (putative centromeres) of the 13 chromosomes of *A. bisporus* var. *bisporus* homokaryon H97. Each colour represents a different type of transposable element. The numbers and positions of repetitive elements vary enormously between chromosomes.

Figure S5. Alignment of LRCs (putative centromeres) of homokaryons H39 and H97. For most chromosomes (example shown of chromosome 2) alignment of the LRCs shows that number and positions of repetitive elements vary considerably. Exemptions are the LRCs of chromosomes 5, 6, 9 and 10. This might suggest that H39 and H97 share a common ancestor.


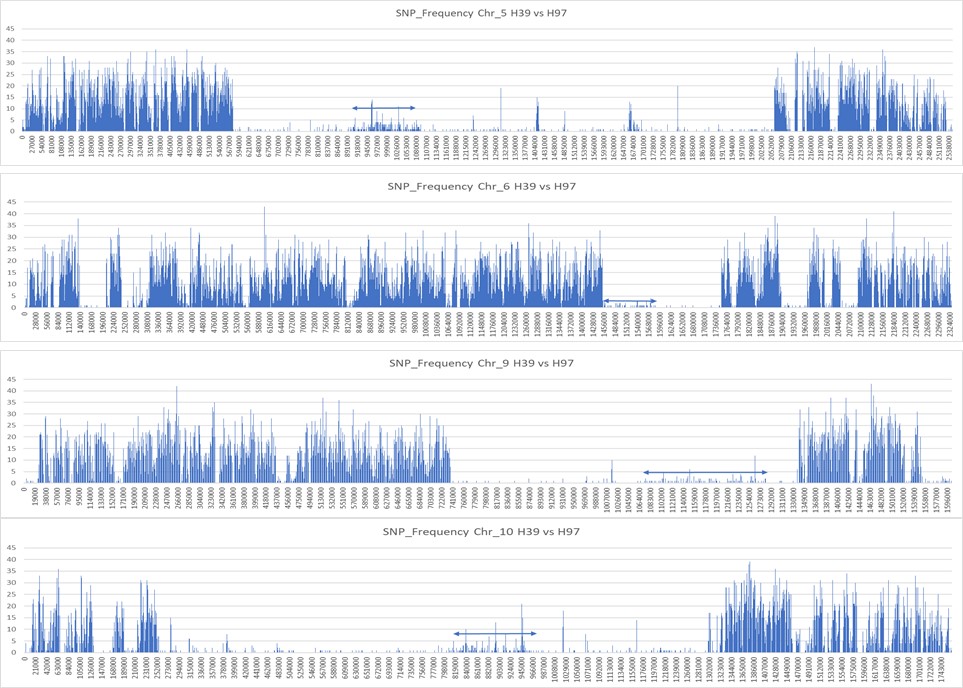


Figure S6. SNP frequency distribution of H39 vs H97 along chromosomes 5, 6, 9 and 10. These chromosomes have clearly very similar sequences indicated by regions with a very low SNP frequency. The position of the LRC (putative centromere) is indicated by an arrow. These LRCs are located in regions with a slightly higher SNP frequency than their surroundings.

Figure S7. A: Alignment of part of the LRC and the left flanking region for chromosome 1 and 2 of H97 and H119p4. The alignment shows clearly that the flanking regions align well whereas the LRC region lack any collinearity. B: Integrative Genomic Viewer of the gene model, repeats and methylation tracks of the same regions as aligned in A. It shows clearly that methylation is restricted to the LRC region.

Figure S8. Alignment of the helicase core region of non-subtelomeric (upper two sequences) and subtelomeric RecQ helicases (lower 2 sequences) showing the typical motifs conserved in helicases ^35^

Table S1. Statistics on whole genome sequences and annotation.

Table S3. Putative gene models found in LRCs that might not represent repetitive elements. For each homokaryon only a few gene models were found (column 2) and for only 4 genes a putative function could be found (column 5).

|  | Gypsy_1 | |
| --- | --- | --- |
| Position | in centromere | outside centromere |
| # Gypsy copies | 19 | 51 |
| # nucleotides | 64068 | 173094 |
| % deviating from consensus | 4.64 | 1.73 |

Table S4. Gypsy 1 is a LTR_retrotransposon present inside and outside the LRC (putative centromere). The table shows that the variation in sequence (# nucleotides deviating from the consensus sequence) is higher for copies within the LRC than outside, indicating that the mutation rate is higher within the LRC than elsewhere in the genome.

1 Inglis, P. W., Rigden, D. J., Mello, L. V., Louis, E. J., & Valadares-inglis, M. . Monomorphic subtelomeric DNA in the filamentous fungus, metarhizium anisopliae,contains a RecQ helicase-like gene. *Molecular Genetics and Genomics* **274**, 79-90, doi:<http://dx.doi.org.ezproxy.library.wur.nl/10.1007/s00438-005-1154-5> (2005).

2 Kumar, S., Stecher, G., Li, M., Knyaz, C. & Tamura, K. MEGA X: Molecular Evolutionary Genetics Analysis across Computing Platforms. *Molecular Biology and Evolution* **35**, 1547-1549, doi:10.1093/molbev/msy096 (2018).
